# Supplementary material for: Drug delivery from a solid formulation during breastfeeding—A feasibility study with mothers and infants
Source: PLoS One. 2022 Mar 4;17(3):e0264747. doi: 10.1371/journal.pone.0264747 (PMC8896718; doi:10.1371/journal.pone.0264747)
Supplement: S1 Table — (DOCX) [file pone.0264747.s002.docx]

**S1 Table. Comparison of maternal expectations before and experiences after vitamin delivery during breastfeeding using a commercial nipple shield.**

|  | Before study feed | After study feed | Change [%] | Quote of expectation |
| --- | --- | --- | --- | --- |
| The nipple shield with a vitamin tablet… | | | | |
| …will be/was easier than using an oral syringe. | 7.0 ± 1.6 | 8.3 ± 1.8 | +19 | - They are upset, but you need to give the medicine first. So then you have to get that all sorted out. Then they are crying, because they are hungry, and by the time you actually start to feed, they are already really distressed. Whereas I guess, if you can do it all in one go, then that is just going to be a bit easier. (M9, NS) - […] you are doing breastfeeding anyway. Hopefully it will be less messy. There is no sort of error with calculating, mismeasuring with the syringes. You have just one tablet and that’s the dose. (M2, NS) - Because you are already breastfeeding, it makes it a bit simpler in that sense. That it’s faster with what you are already naturally anyways doing. (M8, no NS) |
| …will make/ made me less worried. | 7.2 ± 2.0 | 8.6 ± 1.5 | +19 | - … I think it would be positive for the mother, if it is positive for the baby. […] So I am just making the assumption it will be nicer for [my baby] and in turn it will be nicer for me. (M6, NS) - Probably [I’d be] more confident in giving medicine, to be honest. Because then it’s not having to stress him out, nor do I stress me out. (M10, NS) - I would probably feel more comfortable doing it this way, just ‘cause it’s something we are already doing. (M17, NS) |
| …will make/ made my baby feel less upset/ distressed. | 7.7 ± 1.5 | 8.6 ± 1.4 | +12 | - I think, if you are able to give your baby medication in a more natural way where it is just part of their routine anyway, I think that’s good. I think it’s less stressful. (M12, no NS) - I think it’s a good idea, ‘cause it’s a way of them getting the medication that’s doing something they would normally do. You are not forcing them. It’s a natural process. (M20, no NS) - I imagine the nipple shield would be less stress for the baby. Because it’s part of a regular interaction. Whereas a syringe would be something completely new, external coming in. (M16, no NS) - I guess, if baby is already familiar with a nipple shield, it shouldn’t notice the difference of receiving something else as well. So I guess that would be the least invasive way of delivering. If a baby is not familiar with a nipple shield, then I don’t know if it would be as smoothly received. Because that’s another thing that the baby has to adapt to, that would change. (M16, no NS) |
| …will help/ helped me to feel closer to my baby. | 7.7 ± 1.6 | 8.4 ± 1.7 | +9 | - I would probably going to be closer to him and it would feel more like a natural part of the feed, rather than kind of going away, taking the medicine out of a bottle, putting it in a syringe, and then feeding it to him. (M9, NS) - I don’t know if it really changes closeness, because it is such a short… it’s such a snapshot of time. (M4, no NS) |
