# Supplementary material for: Drug delivery from a solid formulation during breastfeeding—A feasibility study with mothers and infants
Source: PLoS One. 2022 Mar 4;17(3):e0264747. doi: 10.1371/journal.pone.0264747 (PMC8896718; doi:10.1371/journal.pone.0264747)
Supplement: S2 Table — (DOCX) [file pone.0264747.s003.docx]

**S2 Table. Summary of maternal experience and assessment of breastfeeding during vitamin B12 delivery via a nipple shield whilst feeding.**

|  | Agree-ment [%] | Strongly agree [%] | Quote |
| --- | --- | --- | --- |
| The nipple shield with a vitamin tablet… | | | |
| …was a positive experience. | 95 | 45 | - I think it surpassed my expectation. Mainly because I was slightly concerned about using this silicone and having the tablet, and how it would all work. Whereas now that I have done it, I can see that it is actually quite a natural process. […] I wish I’d be able to administer all medicines like that rather than using syringes. (M5, no NS) - It was pleasantly surprising, really. […] You know, it was just an idea before, and now having done it, I feel good about it. I would say that it exceeded my expectations. (M1, NS) - I think after doing it, using the shield, and having him breastfeeding normally on it, I’d say it was a really positive experience. […] I really think it is a more natural way to deliver the vitamin. (M4, no NS) |
| …was easy to use. | 95 | 65 | - […] this was just such a smooth, easy process. (M5, no NS) - It was really easy to put on. (M7, no NS) - […] I didn’t feel that I was giving him medicine at all. (M6, NS) - I think this method of being able to give medication is so much easier. […] You are not having to use a syringe necessarily to give the medication, which can be quite difficult. […] It’s part of what would have been your everyday routine anyway, instead of having to include something that’s not necessarily something that they would want to do. (M20, no NS) |
| …was comfort-able to wear. | 95 | 65 | - I thought it might be… I don’t know awkward or difficult. I thought the shield might slip off, or something. But it was actually very comfortable. (M12, no NS) - How comfortable the shield was for me […] - I was quite surprised. (M5, no NS) - You know, I was aware that there was a layer in between. But then it was less painful for me - the whole idea of the nipple shield is to ease the pain of the nipple, so that actually made it more comfortable anyway. (M4, no NS) - [Breastfeeding] is a bit different with a shield, ‘cause you don’t necessarily get the same contact. But I think for the benefits it’s not enough to worry about. Because the benefits far outway that. (M20, no NS) |
| My baby…. | | | |
| …latched as usual. | 95 | 65 | - I was surprised how easily he still latched, and that he didn’t even notice that there was a nipple shield in the way. (M7, no NS) - I think he thought it was a bit strange at first because he has not had to use a breast shield before. So it was obviously a different texture to what he was used to. But once he got used to it, it didn’t stop him at all. […] it [also] hasn’t stopped him then going onto the other side, which didn’t have the breast shield on – which is a positive. (M20, no NS) |
| …breast-fed as usual. | 90 | 65 | - I couldn’t tell any difference between feeding him with the tablet in there or without. […] it was completely flawless, it just worked perfectly. (M6, NS) - I was expecting more hurdles there. But there weren’t any. She did very well. She didn’t have any problems with taste or anything changing. […] I think that was my main concern initially: How would she react to it having the change? And she was absolutely comfortable with it. (M8, no NS) - That surprised me the most, that he just didn’t seem bothered. (M5, no NS) |
