# Supplementary material for: Drug delivery from a solid formulation during breastfeeding—A feasibility study with mothers and infants
Source: PLoS One. 2022 Mar 4;17(3):e0264747. doi: 10.1371/journal.pone.0264747 (PMC8896718; doi:10.1371/journal.pone.0264747)
Supplement: S3 Table — (DOCX) [file pone.0264747.s004.docx]

**S3 Table. Summary of maternal acceptability of medicine and nutrient delivery during breastfeeding using a commercially available ultrathin contact nipple shield whilst feeding.**

|  | Agree-ment [%] | Strongly agree [%] | Quote |
| --- | --- | --- | --- |
| I prefer to give medicines/ nutrients using a nipple shield over using an oral syringe. | 85 | 30 | - It would definitely be something I would consider doing over a syringe, if I had the option. (M17, NS) - Just because it’s natural, it’s the best. That’s how she is been giving her food. (M12, no NS) - It’s because [breastfeeding is] what she’s used to. And that means there is not gonna be a traumatic experience. It’s not gonna be something scary or something that she doesn’t know, that she doesn’t understand what’s going on. Which is always helpful […] especially when the baby is poorly you don’t want to add more stress to the whole procedure. (M19, NS) - To have something simple as that…then I would always choose the nipple shield, and that option. (M6, NS) - Whether I would use it all the time, I don’t know. […] I might use a combination of ways. But definitely the fact that it caused him less stress having it, was a major plus. […] I am glad I tried this way as well. (M4, no NS) - It has not been a bad experience at all. But I just don’t know if it is the preferred method for me. (M11, no NS) |
| I think the nipple shield could be an acceptable method for nutrient delivery. | 100 | 65 | - I would say vitamins would be more preferable, because medicine it depends on how distressed the baby is. Because sometimes you have to hold them and give them the medicine. They are not interested in feeding, they are not feeling that well, and everything. And then it could become a bit hard. (M8, no NS) - I think anything you can save a baby from having to kind of have to have syringes and things like that. (M6, NS) - I think, as long as it’s proofed that they absorb the amount they need to absorb, I wouldn’t have an issue with it being medication as well at all. If anything it’s less stressful, so […] you are actually more likely to get the full amount of it [delivered] in this scenario. (M20, no NS) - It could be anything, as long as there is a way to know that he has definitely got everything. Which… It was really easy to tell, that the tablet had completely dissolved, and therefore he had taken the whole tablet. So it’s just making sure he got the right dose. (M7, no NS) |
| I think the nipple shield could be an acceptable method for medicine delivery. | 95 | 60 |  |
| I would like that medicine/ nutrient delivery during breastfeeding becomes possible for parents in the future | 100 | 55 | - I think it would be good, if it is possible. As long as there is proper training on how to give the medicine. But then you have to have training on how to give a syringe anyway. So it shouldn’t make much difference, it’s just a different way of doing it. Similar, but in a less invasive way. (M7, no NS) - It’s very intimate with the baby. […] You are not imposing anything on the baby, it’s not an aggressive method of delivery. I think if someone is breastfeeding, this should definitely be presented as an option, as a way to deliver the medicine or the nutrient supplement. (M16, no NS) - I think it is a perfectly good option. Just ‘cause I don’t particularly get on with the shield, I think for other mums, […] it would be a good idea for some. [...] I think options are always a good idea. (M11, no NS) - It would be much easier in the future if that’s a method. Because the syringe… yes, you have the odd child that likes the syringe, but not every child does. It varies quite a lot. (M20, no NS) - I think it is more of a personal choice. It would always come down to having a choice there. But having a choice is good. (M8, no NS) |
