# Supplementary material for: Drug delivery from a solid formulation during breastfeeding—A feasibility study with mothers and infants
Source: PLoS One. 2022 Mar 4;17(3):e0264747. doi: 10.1371/journal.pone.0264747 (PMC8896718; doi:10.1371/journal.pone.0264747)
Supplement: S1 File — (DOC) [file pone.0264747.s005.doc]

Study Title: The feasibility of drug delivery to infants during breastfeeding (FEDD)

Date and Version No: February 25th 2018, Version 1

| Principal Investigator: | Dr Kathryn Beardsall, Addenbrooke’s Hospital  Email: kb274@medschl.cam.ac.uk  Telephone: 01223 746791 |
| --- | --- |
| Investigator: | Miss Theresa Maier, University of Cambridge  Email: tm520@cam.ac.uk  Telephone: 07778627858 |
| Sponsor: | University of Cambridge and University of Cambridge Addenbrookes Hospital NHS Trust |

AMENDMENT HISTORY

| **Amendment No.** | **Protocol Version No.** | **Date issued** | **Author(s) of changes** | **Details of Changes made** |
| --- | --- | --- | --- | --- |
|  |  |  |  |  |

# SYNOPSIS

| **Study Title** | The feasibility of drug delivery to infants during breastfeeding (FEDD) |
| --- | --- |
| **Study Abstract** | **Background:**  Current methods of oral infant drug delivery are limited, and confront parents with numerous challenges. In developing countries oral syringes and spoons are inadequate due to limitations of clean water, and parents in developed countries have reported particular emotional and practical challenges in handling devices such as oral syringes. To overcome these challenges the Department of Chemical Engineering and Biotechnology has been working on the development of a Therapeutic Nipple Shield, enabling medicine and nutrient delivery during breastfeeding. It consists of a nipple shield device with a therapeutic insert, capable of releasing medicine/nutrients into human milk during the feed. A qualitative descriptive study performed in 2016 at the Addenbrooke’s Hospital/Rosie Hospital encouraged the translation of this therapeutic delivery concept into a clinical setting.  **Aims:**  This study aims to assess the feasibility of medicine and nutrient administration to infants during breastfeeding.  **Methods:**  This is a prospective observational study of ‘drug’ delivery during breastfeeding. A Medela ultrathin contact nipple shield combined with vitamin B12 as a tracer during a standard breast feed will be used to determine feasibility of ‘drug’ delivery. Successful delivery will be quantitatively assessed through increase in vitamin B12 level post feed. Secondary outcomes will include maternal expectation experience and acceptability, which will be evaluated through pre- and post-delivery semi-structured open-ended interviews. |
| **Internal ref. no.** |  |
| **Study Design** | Interventional Study Design: Single centre feasibility study  Study Classification: Utility |
| **Study Participants** | Breastfeeding infants up to 12 months of adjusted age and their mothers |
| **Planned Sample Size** | 30 infant mother pairs (i.e. 30 infants and 30 mothers) |
| **Follow-up duration** | None |
| **Planned Study Period** | Recruiting and study period: 12 months starting on 1 April 2018 |
| **Primary Objective**  (referred to as  “objective 1”): | *Objective 1:*  Efficacy: Quantitative change in infant serum vitamin B12 concentration |
| **Secondary Objectives**  (referred to as  “objective 2”): | *Objective 2.1*: Qualitative impact on breastfeeding behaviour of infant and/or mother  *Objective 2.2*: Parents’ perspective on proposed technology |
| **Primary Endpoint** | Change in vitamin B12 concentration post feed |
| **Secondary Endpoints** | Qualitative assessment of impact on maternal expectation, experience and acceptability. |
| **Intervention (s)** | Delivery of vitamin B12, during breastfeeding using a Medela ultrathin contact nipple shield. |

# BACKGROUND AND RATIONALE

Need for the development of an infant drug delivery system

According to the World Health Organization (WHO), pneumonia, diarrhoea, malaria, meningitis, tetanus, HIV and measles account for more than fifty percent of the 5.9 million child deaths worldwide before the age of five, 16,000 every day. Most of these child deaths could be prevented or treated with access to simple and affordable interventions.^1^ Yet, current methods of infant drug delivery are often inadequate due to clean water, sterilization, and the therapeutics’ shelf-life requirements, with additional need of refrigerated storage for liquid formulations.^2^ These problems limit the availability of existing technologies in many developing countries.

In developed countries, many conventionally available drug delivery systems are not primarily designed for paediatric patients.^3^ Lopez *et al.* emphasize that age-appropriate paediatric administration devices would have the potential to significantly improve the acceptability of pharmaceutical products.^4^ Currently available paediatric drug delivery technologies, such as drinking bottles, dosing cups/spoons, oral syringe and dropper, medicine dummy, and the Supplementary Nursing System are limited in their applicability in resource-limited settings, due to potable water, sterilization and refrigeration requirements (particularly a challenge in developing countries), unpalatability, and the potential presence of harmful excipients.^9–11^ Independent of the country’s development state, liquid formulations often lead to dosing errors.^12^ Moreover, with exception of the Supplementary Nursing System none of the above mentioned devices can be used during breastfeeding. As 36% of infants aged 0 − 6 months worldwide are exclusively breastfed without the use of formula supplementation, an oral drug and nutrient delivery system applicable for the breastfeeding infant population is critically needed.^5^

Breast milk is also often referred to as “a personalised medicine for infants”, based on its nutritional and immunological characteristics.^6^ A delivery device used during breastfeeding would have the additional advantage of advocating and facilitating the beneficial practice of breastfeeding in both high-income and low-resource settings. Past research has shown that children breastfed for longer periods have a decreased infectious morbidity and mortality, fewer dental malocclusions, higher intelligence, as well as a lower risk of obesity and non-communicable diseases, such as asthma and type 2 diabetes, in later life.^6–8^ According to Victora *et al.*, the scaling up of breastfeeding alone, i.e. without addition of any medicine/nutrient supplement, could prevent 823,000 annual deaths in children under the age of five.^6^

Development of an infant drug and nutrient delivery system (*in-vitro* prototype)

In 2008, the Department of Chemical Engineering responded to a WHO-led call for new medication delivery technologies by developing an infant drug and nutrient delivery system, referred to as a “Therapeutic Nipple Shield (TNS). It comprises a nipple shield device containing a therapeutic (medicine/nutrients), and allows therapeutic delivery during the process of breastfeeding. During a feed the therapeutic is released from the shield and delivered to the suckling infant with the flow of human milk. In the past, different designs were investigated *in-vitro*: a modified nipple shield, referred to as the NSDS, enabling the positioning of a rapidly disintegrating tablet or other solid dosage form within a “pocket-like” space within the upper part of the silicone nipple, as well as commercially available ultrathin contact nipple shields.

Breastfeeding simulation apparatus

To ease *in-vitro* testing of prototypes (modified nipple shields) and therapeutic-loaded inserts, often referred to as API (active pharmaceutical ingredient)-loaded inserts, a breastfeeding simulation apparatus was developed by Gerrard *et al.*, simulating both the process of lactation and infant feeding.^11^ The apparatus is capable of resembling average flow rates/patterns of milk during breastfeeding, as well as of mimicking, monitoring and recording the suckling pressure of infants.^11^ Within the apparatus, milk is heated to a physiological relevant temperature and delivered through a silicone human breast mimic.^11,13^ To date, extensive proof of concept studies using the simulation apparatus have been conducted, establishing the feasibility of the drug and nutrient delivery system to deliver model compounds using various dosage forms into breast milk.^11^

Solid dosage forms (*In-vitro* research)

From 2013 to 2016, research by Scheuerle *et al.* focused on the manufacture of paediatric rapidly disintegrating tablets through direct compression, and the analysis of their disintegration characteristics.^14^ In addition, the delivery of zinc sulphate from tablet formulations into breast milk was investigated.^15,16^ As rapidly disintegrating tablets are designed to disintegrate completely in the mouth before swallowing,^14^ they were believed to possess promising characteristics for administration using the drug and nutrient delivery system. Current results however reveal that - despite formulation optimisation studies in collaboration with the University College London School of Pharmacy - the time required for the disintegration of rapidly disintegrating tablets in human milk exceeded the time of an average breastfeed by almost two fold.^15^ Research by Gerrard *et al.* investigated the delivery of sodium dodecyl sulphate (SDS) via non-woven fibre inserts aiming at preventing HIV transmission from mother to child.^17,18^

Qualitative descriptive study

To evaluate parents’ attitude towards a nipple shield device capable of administering medicine and nutrients during breastfeeding, two scoping exercises were conducted. Thereby, exploratory interviews in Kenya evaluated the suitability of the TNS for delivery of antiretroviral prophylaxis for the prevention of HIV mother-to-infant-transmission, focusing on acceptability respective to its practicability and cultural context.^19^ In this low-resource setting, use of a TNS was considered “potentially acceptable”, requiring careful consideration of the respective cultural setting and provision to enable sustainable access.^19^ To determine the feasibility of a nipple shield delivery device in resource-rich environments, and to specifically identify potentially required device changes, an additional scoping exercise was conducted by the (Principal) Investigator from May to July 2016 at the Addenbrooke’s Hospital in Cambridge/UK. Questions aimed at (a) exploring problems of oral drug administration anticipated by parents, (b) assessing mothers’/fathers’ experience with commercially available nipple shields - a device similar to the drug and nutrient delivery system,
(c) identifying preferred design characteristics, (d) identifying potential challenges for the realisation of the clinical investigation and mitigation strategies. Hereby, parents and nurses’ felt that a TNS could foster mother-infant bonding and encourage parental empowerment in high-resource settings, particularly in special care environments. They all supported the delivery of vitamins during breastfeeding using the TNS. With regard to the TNS’ design, parents and healthcare professionals raised several concerns about cleanliness, fit, and practicability of a custom-made nipple shield, while some parents assumed easier handling of a commercially available device already used for normal feeding. As a result, the use of a Medela ultrathin contact nipple shield was preferred over a modified nipple shield prototype used for previous *in-vitro* testing at the Department of Chemical Engineering and Biotechnology, University of Cambridge. At the same time parents and healthcare professionals supported our objective of conducting a feasibility study, in which a therapeutic is delivered to breastfeeding infants from a Medela ultrathin contact nipple shield (*in-vivo evaluation).*

Conceptual considerations for conducting a clinical investigation

To proof the capability and feasibility of medicine and nutrient administration to infants during breastfeeding, the following components are required:

*1. A nipple shield device that can be used during breastfeeding:* Based on the result obtained as part of the qualitative descriptive study conducted from May to July 2016 with parents and staff through convenience sampling on a level 3 Neonatal Intensive and a Transitional Care Unit of the University of Cambridge Addenbrooke’s Hospital NHS Trust, a commercially available Medela contact ultrathin nipple shield will be used. Three different sizes will be available to address both maternal and infant physiology (16 mm, 20 mm, 24 mm).

2. *An in-vivo tracer in form of a suitable oral dosage form: T*he *in-vivo* tracer of choice is vitamin B12, being of great importance for the production of red blood cells, as well as the development of the central nervous system.^20–22^ For the proposed study, commercially available 1000 µg Methylcobalamin Vitamin B12 Tablets (Just Vitamins Ltd, UK) will be used as the therapeutic insert for the Medela ultrathin contact nipple shield. The sublingual tablets are suitable for vegetarians and vegans, and do not contain any known allergenic components. Lab-based research using an apparatus to simulate breastfeeding has shown slow release of these tablets, ensuring that vitamin B12 will be appropriately mixed with human milk (data can be found in the appendix of this protocol). Although the daily recommended reference intake for infants up to 6 months of age is 0.5 μg, vitamin B12 has no toxic effects. Research in adults has shown that from a 1000 μg dose, only about 1.5 - 2.0 μg are uptaken by active absorption plus about 10 - 30 μg by passive diffusion.^23^ Moreover, a recommended I.M. Schilling test flushing dose of 1000 μg I.M. is used for both adults and infants to treat vitamin B12 deficiency.^24^ The World Health Organisation states that “intake of 1000 µg vitamin B12 has never been reported to have any side-effects”.^23^ Suitability was also confirmed by a previous publication reporting the use of 400 μg I.M. hydroxycobalamin in infants as young as 3 weeks (Torsvik, I. et al. (2013). Cobalamin supplementation improves motor development and regurgitations in infants: results from a randomized intervention study. The American Journal of Clinical Nutrition, 98(5), 1233-1240.), as oral delivery of 1000 µg will result in an uptake lower than the I.M. dose used. Throughout the feed vitamin B12 will be released into the human milk by the breastfeeding infant. Successful delivery will be quantitatively assessed through blood analysis 6-8 hours after administration. A blood sample, taken within a week before administration, will serve as the baseline for each infant.

# OBJECTIVES

## 3.1 Primary Objective

*Objective 1*:

Feasibility and efficacy: Quantitative assessment, of the change in vitamin B12 concentration in the infants’ blood following vitamin B12 delivery.

## 3.2 Secondary Objectives

*Objective 2.1*: Identification of potential qualitative impact on breastfeeding behaviour of infant and/or mother

*Objective 2.2*: Identification of parents’ perspective on technology

# STUDY DESIGN

**4.1 Summary of Study Design**

This is a single centre feasibility study that will recruit mother and infant pairs from the Addenbrookes Hospital. The study will be undertaken at Addenbrooke’s Hospital Hospital. This study involves approaching parents about potential study participation while their infant is in impatient therapy. There will be a reasonable time (at least 24 hours) for parents to read all the information regarding study participation, to ask questions, and to decide about participation as they feel appropriate. The study will involve the delivery of commercially available vitamin B12 tablets (Just Vitamins Ltd, 1000 µg vitamin B12 as Methylcobalamin) using a commercially available Medela ultrathin contact nipple shield during breastfeeding (aim: 5-10 minutes of breastfeeding). Two infant blood samples, the first one within a week before vitamin B12 delivery, the second one 6-8 hours following delivery, will be obtained by trained healthcare staff, preferable at the same time as clinical samples are taken. Before and after the delivery of vitamin B12 from an ultrathin contact nipple shield, mothers will be interviewed to evaluate their expectation, experience and acceptability. Choice with regards to preferred method of breastfeeding with a nipple shield will be respected, as well as that of a mother to decline to participate. A total of 30 mothers and their breastfeeding infants will be recruited.

## 4.2 Primary and Secondary Endpoints/Outcome Measures

Primary Endpoint:

Detection of the change in vitamin B12 concentration in the infants’ blood 6-8 hours following vitamin B12 delivery

Secondary Endpoint:

Qualitative assessment of impact on maternal feeding expectation, experience and acceptability

**4.3 Study Participants**

## 4.3.1 Overall Description of Study Participants

The proposed research is a single centre (NHS Addenbrooke's Hospital) feasibility study. All breastfeeding mothers with an inpatient infant of up to 12 months of adjusted age are eligible for inclusion in the study. The sample size for the research consists of 30 infants and their mothers, aiming to provide a big enough sample size to ensure a representative and reliable outcome. It was determined based on recommendations by Billingham *et. al*, analysing 79 studies from the United Kingdom Clinical Research Network (UKCRN) database.^25^

## 4.3.2 Inclusion Criteria

Recruiting for study participants

- Mother
  - No known allergy or hypersensitivity against any ingredient of the commercially available Methylcobalamin Vitamin B12 Tablets (Just Vitamins Ltd, UK) used in the study
- Infant
  - Aged between 7 days and 12 months of adjusted age
  - Established breastfeeding (exclusively or non-exclusively)
  - No known allergy or hypersensitivity against any ingredient of the commercially available Methylcobalamin Vitamin B12 Tablets (Just Vitamins Ltd, UK) used in the study

**4.3.3 Exclusion Criteria**

The participant may not enter the study if ANY of the following apply:

- Breastfeeding not well established
- Infant not feeding properly
- Allergy or hypersensitivity against any ingredient of the commercially available Methylcobalamin Vitamin B12 Tablets (Just Vitamins Ltd, UK) used in the study (infant or mother)
- Medical conditions that could negatively influence swallowing, and thus breastfeeding.

## 4.4 Study Procedures

**4.4.1 Recruiting Procedure**

Infants who fulfil eligibility criteria will be identified by the medical and nursing staff based on their date of birth/medical record. Parents/mothers will be approached by the healthcare team, and if the parents/the mother are/is happy to receive information about the study, the research team will speak to the family and provide written information about the research study. Written information sheets will be provided to the parents/mothers with sufficient time (at least 24 hours) to read and ask questions. If a mother would like to participate with her infant, a time/day suitable for the mother will be scheduled either as an inpatient or to return at a time convenient to the family

**4.4.2 The ‘delivery system’**

For the proposed clinical study, a commercially available Medela ultrathin contact nipple shield will be used, as well as a therapeutic, which can serve as an *in-vivo* tracer by enabling detection in the infant’s blood following delivery. The *in-vivo* tracer and the nipple shield device to be used for the study, are described in more detail below.

Use of vitamin B12 as an in-vivo tracer:

The study involves the oral delivery of vitamin B12 from commercially available sublingual tablets (1000 µg), chosen based on their suitability for vegetarians and vegans, and their absence of any known allergenic ingredients. Although the manufacturer states that excessive consumption of the tablets may produce laxative effects, vitamin B12 has no toxic effects, and a dose of 1000 µg I.M. is clinically used as the Schilling test flushing dose for both adults and children.^24^

To investigate the vitamin B12 tablets' disintegration properties, lab-based studies were performed. Hereby, the vitamin B12 tablets were placed in the silicone teat of a 20 mm Medela ultrathin contact nipple shield, and delivery into full-fat cow's milk investigated in triplicate using a breastfeeding simulation apparatus, capable of simulating both the process of lactation and infant feeding by mimicking the average flow rates/patterns of milk during breastfeeding and the infant’s suckling pressure at physiological relevant conditions. The percentage of tablet released within 5, 10 and 20 min was approximately 41%, 62%, and 74% respectively. No tablet break-offs during disintegration or dislocation of the remaining tablet through the silicone teat's three holes following the experiment was observed. Data is provided in the appendix.

Use of a commercially available Medela ultrathin contact nipple shield:

A commercially available Medela contact ultrathin nipple shield of either 16 mm, 20 mm, or 24 mm size will be used for vitamin B12 delivery. While maternal responses to the use of nipple shields is diverse, publications have shown that there is no significant change in infant breast milk consumption for a feed with and without Medela contact ultrathin nipple shields, while even enabling an increase in milk transfer to preterm infants.^26,27^

**4.4.2 Informed Consent**

Written informed consent by the mother for her and her infant's participation in the study will be obtained on the study day prior to any study related procedures being undertaken. The mother and her infant can withdraw from the study at any point.

**4.4.3 Formal Assessment**

After written informed consent is obtained:

1. A blood sample will be taken from the infant within a week before vitamin B12 administration. The identified blood vitamin B12 concentration before vitamin B12 delivery will serve as the baseline concentration for each infant. The sample will be frozen until analysis.
2. The mother will participate in a pre-intervention interview to describe her current thoughts and expectations about the study, as well as to provide details about recent breastfeeding practice. The interview will be recorded using digital voice recording software for later analysis, transcribed verbatim, and potentially identifiable data anonymized.
3. The *in-vivo* tracer vitamin B12, in form of a commercially available sublingual vitamin B12 tablet formulation, will be delivered to breastfed infants from a Medela ultrathin contact nipple shield during a normal feed. Throughout the feed, vitamin B12 will be released into the human milk and consumed by the breastfeeding infants.
4. The mother will complete a *post-*intervention interview, providing information about
   i) her experience (acceptability) of the delivery system, ii) potential changes of the infant’s breastfeeding behaviour, iii) differences to expectations indicated in the *pre-*intervention study interview, iv) areas of improvement. The interview will be recorded using digital voice recording software for later analysis, transcribed verbatim, and potentially identifiable data anonymized.
5. An infant blood sample will be taken 6-8 hours following delivery of vitamin B12. The sample will be frozen until analysis.

**4.4.2 Data Collection Study Assessments**

Demographic data about the study participants will be collected at baseline from the mother and from the medical notes. Blood samples will be collected within one week before and 6-8 hours after vitamin B12 delivery. Interviews with mothers will be recorded before and after vitamin B12 delivery - they will be recorded and then transcribed by the research team, and potentially identifiable data anonymized. It is intended to publish the results of this study in a relevant academic journal.

## 4.6 Analysis

Blood samples will be analysed at the at the NHS Addenbrooke's Hospital Cambridge. Interview responses will be evaluated using standard qualitative approaches.

## 4.7 Definition of End of Study

The end of study is the last patient visit.

# Interventions

Delivery of vitamin B12, in form of a commercially available sublingual vitamin B12 tablet formulation, to breastfeeding infants from a Medela ultrathin contact nipple shield during a normal feed.

# STATISITICS

## 6.1 The Number of Participants

It is intended that 30 mothers and 30 infants take part in the study as outlined, aiming at providing a big enough sample size to ensure a representative and reliable outcome. The sample size was determined based on recommendations by Billingham *et al.* analysing 79 studies from the United Kingdom Clinical Research Network (UKCRN) database.^28^ This is a feasibility study but based on the literature for preterm infants supplementation with B12 between 8-12 weeks led to an approximate 3 fold rise in serum B12 levels. As such this sample size would provide 90% power at the 5% level to demonstrate a 2 fold increase in B12.

# Ethics

## 7.1 Participant Confidentiality

The study staff will ensure that the participants’ anonymity is maintained. The participants will be identified only by initials and a participants ID number on the CRF and any electronic database. All documents will be stored securely and only accessible by study staff and authorised personnel. The study will comply with the Data Protection Act which requires data to be anonymised as soon as it is practical to do so.

**7.2 Other Ethical Considerations**

A mother participating in the study will have to provide consent for the participation of herself and her infant. It is believed that the information provided about her infant’s medical conditions/potential hypersensitivity reactions are correct, and that consent is obtained voluntarily.

## 8. FINANCING AND INSURANCE

All costs for the vitamin B12 tablets used in this study, as well as the vitamin B12 analysis of all blood samples taken, will be paid by the Department of Chemical Engineering and Biotechnology, University of Cambridge. The NHS indemnity scheme or professional indemnity will apply. The study is sponsored by University of Cambridge and Addenbrookes Hospital NHS Trust.

# 9. REFERENCES

1. IGME, U. Levels and Trends in Child Mortality: Report 2015. (2015).

2. WHO. Promoting safety of medicines for children. (2007).

3. Kearns, G. L. *et al.* Developmental pharmacology-drug disposition, action, and therapy in infants and children. *N. Engl. J. Med.* **349,** 1157–1167 (2003).

4. Lopez, F. L. & Ernest, T. B. Formulation approaches to pediatric oral drug delivery: benefits and limitations of current platforms. *Expert Opin. Drug Deliv.* **12,** 1727–1740 (2015).

5. World Health Organization, 2017. Infant and young child feeding, Fact sheet N. 342. http://who.int/mediacentre/factsheets/fs342/en/. (accessed 02.01.16).

6. Victora, C. G. *et al.* Breastfeeding in the 21st century: epidemiology, mechanisms, and lifelong effect. *Lancet* **387,** 475–490 (2016).

7. Harder, T. & Bergmann, R. Duration of breastfeeding and risk of overweight: a meta-analysis. *Am. J. Epidemiol.* **162,** 397–403 (2005).

8. Owen, C. G. & Martin, R. M. Does breastfeeding influence risk of type 2 diabetes in later life? A quantitative analysis of published evidence. *Am. J. Clin. Nutr.* **84,** 1043–1054 (2006).

9. Knoppert, D. C. Pediatric Formulations. *Pediatr. Drugs* **11,** 55–56 (2009).

10. WHO. World Health Report 2013. Research for universal health coverage. (2010).

11. Gerrard, S. E., Orlu-Gul, M., Tuleu, C. & Slater, N. K. H. Modeling the physiological factors that affect drug delivery from a nipple shield delivery system to breastfeeding infants. *J. Pharm. Sci.* **102,** 3773–3783 (2013).

12. Walsh, J. & Bickmann, D. Delivery devices for the administration of paediatric formulations: overview of current practice, challenges and recent developments. *Int. J. Pharm.* **415,** 221–231 (2011).

13. Scheuerle, R. L. Development of a Nipple Shield Delivery System for Oral Delivery of Therapeutics to Breastfeeding Infants. *First Year Rep.* (2014).

14. Scheuerle, R. L. *et al.* Characterising the disintegration properties of tablets in opaque media using texture analysis. *Int. J. Pharm.* **486,** 136–43 (2015).

15. Scheuerle, R. L. *et al.* Characterisation of Zinc Drug Delivery from a Nipple Shield Delivery System using a Breastfeeding Simulation Apparatus (in preparation).

16. WHO. WHO/UNICEF joint statement: clinical manWHO, 2004. WHO/UNICEF joint statement: clinical management of acute diarrhoea.agement of acute diarrhoea. (2004).

17. Gerrard, S. E., Baniecki, M. L. & Sokal, D. C. A nipple shield delivery system for oral drug delivery to breastfeeding infants: Microbicide delivery to inactivate HIV. *Int. J. Pharm.* **434,** 224–234 (2012).

18. Bartok, C. J. & Ventura, A. K. Mechanisms underlying the association between breastfeeding and obesity. *Int. J. Pediatr. Obes.* **4,** 196–204 (2009).

19. Hart, C. W. & Israel-Ballard, K. A. Acceptability of a nipple shield delivery system administering antiviral agents to prevent mother-to-child transmission of HIV through breastfeeding. *J. Hum. Lact.* **31,** 68–75 (2015).

20. Adkins, Y. & Loennerdal, B. Mechanisms of vitamin B12 absorption in breast-fed infants. *J. Pediatr. Gastroenterol. Nutr.* **35,** 192–198 (2002).

21. Carkeet, C. *et al.* Human vitamin B12 absorption measurement by accelerator mass spectrometry using specifically labeled 14C-cobalamin. *Proc. Natl. Acad. Sci.* **103,** 5694–5699 (2006).

22. Doscherholmen, A. & Hagen, P. S. A dual mechanism of vitamin B12 plasma absorption. *J. Clin. Invest.* **36,** 1551–1557 (1957).

23. WHO. *Vitamin and mineral requirements in human nutrition*. (Geneva: World Health Organization, 2005).

24. WHO. Human Vitamin and Mineral Requirements Report of a joint FAO/WHO expert consultation Bangkok, Thailand. (2001).

25. Billingham, S. A. M., Whitehead, A. L. & Julious, S. A. An audit of sample sizes for pilot and feasibility trials being undertaken in the United Kingdom registered in the United Kingdom Clinical Research Network database. *BMC Med. Res. Methodol.* **13,** 104 (2013).

26. Chertok, I. R. A., Schneider, J. & Blackburn, S. A pilot study of maternal and term infant outcomes associated with ultrathin nipple shield use. *J. Obstet. Gynecol. Neonatal Nurs.* **35,** 265–272 (2006).

27. Meier, P. P. *et al.* Nipple Shields for Preterm Infants: Effect on Milk Transfer and Duration of Breastfeeding. *J. Hum. Lact.* **16,** 106–114 (2000).

28. Billingham, S. A. M. *et al.* An audit of sample sizes for pilot and feasibility trials being undertaken in the United Kingdom registered in the United Kingdom Clinical Research Network database. *BMC Med. Res. Methodol.* **13,** 104 (2013).

**11.** **APPENDIX**

**Characterisation of the disintegrating properties of “JustVitamins” vitamin B12 tablets using a breastfeeding simulation apparatus**

**11.1. INTRODUCTION**

To investigate the suitability of commercially available vitamin B12 sublingual tablets for the delivery of vitamin B12 during breastfeeding, lab-based proof-of-concept studies using a breastfeeding simulation apparatus were conducted at the Department of Chemical Engineering and Biotechnology, University of Cambridge. Hereby, the disintegration and delivery of vitamin B12 tablets, intended to be used for the clinical study at Addenbrooke’s Hospital/the Rosie Hospital, into fresh whole cow’s milk was investigated. Methods, results, and discussion are illustrated below.

**11.2. MATERIALS AND METHODS**

**11.2.1. Materials**

The nipple shield device used was a commercially available 20 mm Medela ultrathin contact nipple shield, provided from a midwife at Addenbrooke’s Hospital/the Rosie Hospital, while commercially available 1000 µg Methylcobalamin Vitamin B12 Tablets (Just Vitamins Ltd, UK) served as the therapeutic insert.**[[1]](#footnote-2)** Ingredients: Sorbitol, Stearic Acid, Beetroot, Blackcurrant, Magnesium Stearate, Methylcobalamin (Vitamin B12). The blackcurrant flavoured sublingual tablets are suitable for vegetarians and vegans, and do not contain any known allergenic components. Experiments were performed using fresh full-fat cow’s milk (The co-operative).

**11.2.2. Delivery of vitamin B12 into fresh whole cow’s milk**

Experiments were conducted using the breastfeeding apparatus by Gerrard *et al.*, capable of simulating both the process of lactation and infant feeding by mimicking the average flow rates/patterns of milk during breastfeeding and the infant’s suckling pressure (Gerrard et al., 2013).[[2]](#footnote-3) Based on literature about breastfeeding physiology (Black et al., 1998; Geddes et al., 2008; Macias and Meneses, 2011; Moral et al., 2010)[[3]](#footnote-4),[[4]](#footnote-5),[[5]](#footnote-6),[[6]](#footnote-7), and in accordance with previous work conducted using the simulation apparatus (Gerrard and Larson, 2013; Scheuerle et al., 2017)[[7]](#footnote-8),[[8]](#footnote-9), a flow rate of approximately 5.0 mL min^-1^ , a suction frequency of 1 suction/s, and a temperature range of 33.7 - 35.7°C, simulating the likely temperature of human milk in the infant’s mouth, were chosen for all experiments. The pressure range and amplitude were adjusted to values of physiological relevance (Geddes et al., 2008).^4^ Throughout a suck cycle, the intra-oral vacuum increases with the lowering of the infantile tongue from the baseline pressure (mean maximum pressure) between -50 to - 60 mmHg to its peak vacuum (mean minimum pressure) of -110 to -170 mmHg,^4^ while the maternal nipple elongates and retracts rapidly with an elongation in length by up to two fold.[[9]](#footnote-10) The human breast mimic was set to an angle of 30° downwards from the vertical axis. Fractions were collected for a period of 40 s each with a total of 30 fractions, amounting to a total of 20 min. Calibration of the breastfeeding simulation apparatus’ pumps prior to any experimental work being undertaken, as well as monitoring of temperature and pressure throughout the breastfeeding simulation was performed. Delivery of vitamin B12 was conducted in triplicate for 5 min, 10 min, and 20 min operation.

**11.3. RESULTS AND DISUCSSION**

The associated pressure profiles are illustrated in Figures 1, Figure 2, and Figure 3. The weight of each vitamin B12 tablet before and after the experiments, as well as the percentage release are illustrated in Table 1.

While the pressure profiles vary slightly as a result of the tablet being present in different orientations/amounts over time, no significant difference in pressure profile can be observed. The slow release of the vitamin B12 tablets over time makes it possible that vitamin and human milk can mix appropriately. 41% delivery of 1000 µg vitamin B12 indicate that even short durations of breastfeeding will be sufficient for the successful completion of the research study. Moreover, as disintegration using the breastfeeding simulation apparatus cannot simulate infant tongue movement, nor the elongation of the maternal nipple/breast during breastfeeding, enhanced disintegration can be anticipated during in-vivo delivery of vitamin B12 using commercially available Methylcobalamin Vitamin B12 Tablets (Just Vitamins Ltd, UK). Published literature about lab-based research on the impact of infant tongue movement on the disintegration of tablets during breastfeeding confirms this assumption.[[10]](#footnote-11)

**Table 1.** Overview of tablet weight before and after the breastfeeding simulation experiments, as well as percentage of vitamin B12 released. A commercially available ultrathin contact nipple shield (20 mm) was used.

| Duration of experiment | Weight of milk passed through TNS [g] | Weight of tablets before experiment  [g] | Weight of tablets after experiment [g] | Percent of tablet released [%] |
| --- | --- | --- | --- | --- |
| 5 min | 25.49 ± 0.18 | 0.2611 ± 0.0019 | 0.1549 ± 0.0141 | 40.65 ± 5.73 |
| 10 min | 48.94 ± 0.40 | 0.2596 ±0.0022 | 0.0981 ±0.0050 | 62.22 ± 1.95 |
| 20 min | 91.90 ± 0.08 | 0.2598 ± 0.0029 | 0.0677 ± 0.0225 | 73.95 ± 8.44 |


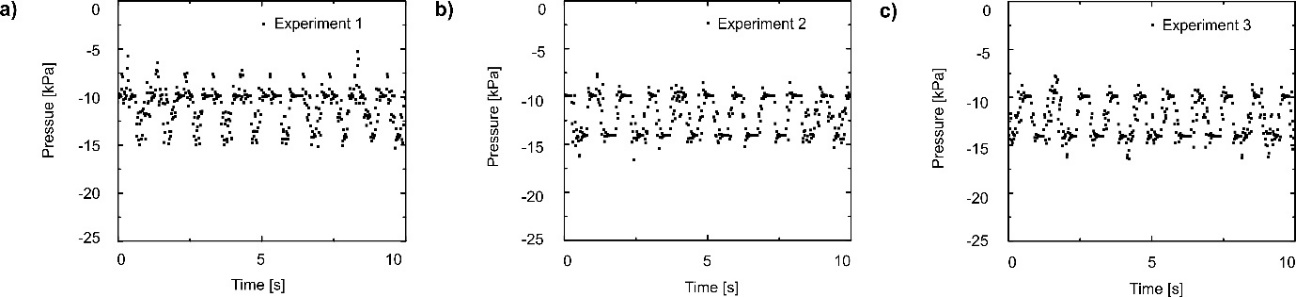


**Figure 1:** Pressure profiles of experiments with a duration of 5 minutes, conducted in triplicate.


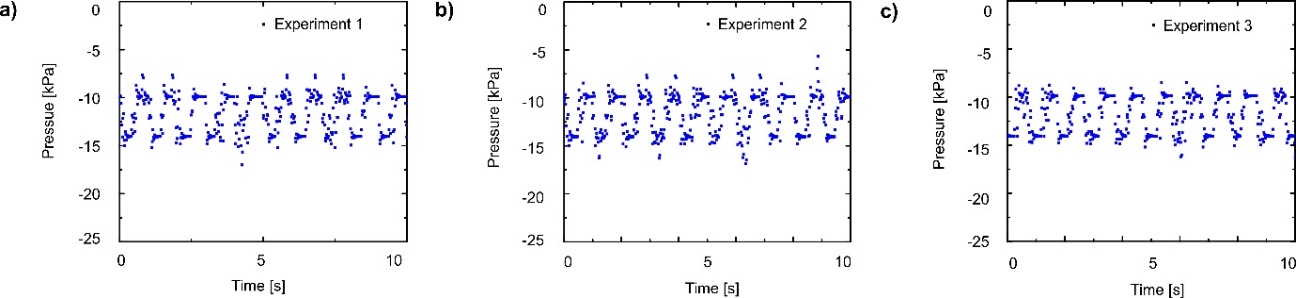


**Figure 2:** Pressure profiles of experiments with a duration of 10 minutes, conducted in triplicates.


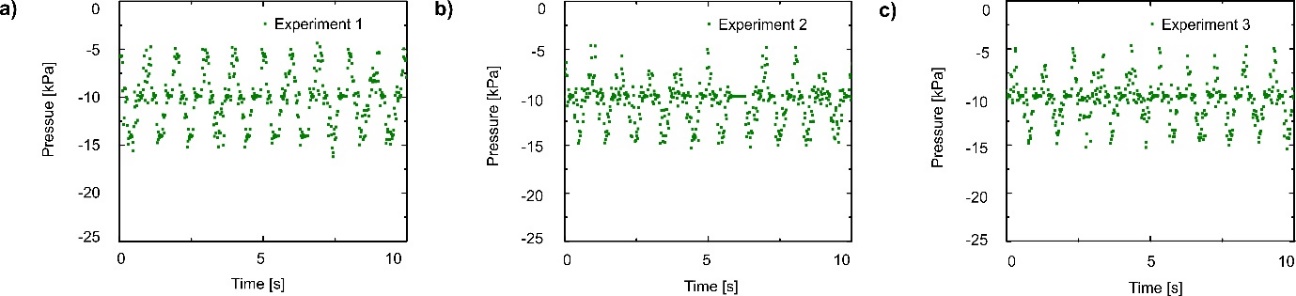


**Figure 3:** Pressure profiles of experiments with a duration of 20 minutes, conducted in triplicate.

**11.4. CONCLUSION**

The breastfeeding simulation results indicate the suitability of 1000 µg Methylcobalamin Vitamin B12 Tablets (Just Vitamins Ltd, UK) as a therapeutic insert for our proposed clinical study with the objective to evaluate the clinical feasibility of the Therapeutic Nipple Shield.

1. [https://www.justvitamins.co.uk/methylcobalamin-vitamin-b12.aspx#.WgMgutVl-Uk](https://www.justvitamins.co.uk/methylcobalamin-vitamin-b12.aspx#.WgMgutVl-Uk). (06/2017) [↑](#footnote-ref-2)
2. Gerrard et al., 2013. *J. Pharm. Sci.* 102, 3773–3783. doi:10.1002/jps.23688. [↑](#footnote-ref-3)
3. Black, 2003, J. Nutr. 133, 1485–1489. [↑](#footnote-ref-4)
4. Geddes et al., 2008. Early Hum. Dev. 84, 471–477. doi:10.1016/j.earlhumdev.2007.12.008. [↑](#footnote-ref-5)
5. Macias et al., 2011. Bol. Med. Hosp. Infant. Mex. 68, 296–303. [↑](#footnote-ref-6)
6. Moral et al., 2010. BMC Pediatr. 10, 1–8. doi:10.1186/1471-2431-10-6. [↑](#footnote-ref-7)
7. Gerrard et al., 2013. Biotechnol. Bioeng. 110, 2058-2062. [↑](#footnote-ref-8)
8. Scheuerle et al., 2017. PLoS One 12, e0171624. doi:10.1371/journal.pone.0171624 [↑](#footnote-ref-9)
9. Black et al., 1998. *The Science of Breastfeeding*. Jones and Bartlett Publishers, 3 edition. [↑](#footnote-ref-10)
10. Scheuerle, et al., 2017. *Journal of pharmaceutical sciences* 106.1 (2017): 193-199. [↑](#footnote-ref-11)
