# Supplementary material for: Drug delivery from a solid formulation during breastfeeding—A feasibility study with mothers and infants
Source: PLoS One. 2022 Mar 4;17(3):e0264747. doi: 10.1371/journal.pone.0264747 (PMC8896718; doi:10.1371/journal.pone.0264747)
Supplement: Attachment — Submitted filename: Response to Reviewers_v15 Nov 2021 stand alone.docx [file pone.0264747.s007.docx]

Dr Kathryn Beardsall

University of Cambridge Associate Professor

University of Cambridge Department of Paediatrics

Cambridge Biomedical Campus

CB2 0QQ

Dear Jamie Males,

**Re: Drug delivery from a solid formulation during breastfeeding – a feasibility study with mothers and infants: Manuscript Number: PONE-D-20-38364R1**

All the changes made to the manuscript are indicated via tracked changes. A detailed response to the reviewers’ comments can be found on the following page.

We hope that the changes made are to your and the reviewers’ complete satisfaction.

**Reviewer 1**

**Minor point:** Line 114 – Indicate the statistical testing method which achieves 90% power.

**Response:** Thank you we have added (two sided superiority) to clarify.

**Reviewer 2**

**Minor point 1:** In the marked up manuscript line 230-232,

a) "Given the small sample size, no conclusive statement can be made, and further investigations would be required but the findings are in keeping with the literature on B12.", can you please specify "no conclusive statement can be made" on what?

**The text was amended to**

“Given the small sample size, no conclusive statement can be made about the interdependency between postnatal age and vitamin B12 absorption, and further investigations are required.”

b) Also please specify e.g. "keeping with the literature on B12 absorption in infants" and provide literature references.

**References were added as requested**: “This is a feasibility study but based on the literature for preterm infants’ supplementation, in which B12 between 8-12 weeks led to an approximate three-fold rise in serum B12 levels (9,10).”

**Minor point 2:** The authors have commented that oral syringe would need to be sterilised which is a disadvantage in LMICs. Would the nipple shield need to be sterilised before use?

**Response:** The initial suggestion was for a disposable or biodegradable nipple shield to avoid the need for sterilization but given that this was a proof of principle in a level 3 unit in the UK we thought this distracted from the main message of the paper.

**Minor point 3:** This sentence needs to be written clearer "While many formulations include taste advancements, masking ingredients are generally avoided in the neonatal population and there may be benefits from this route and the masking effects of milk on the taste of the medication." For example "While many paediatric formulations include taste masking techniques, excipients used in the neonatal population should be limited. There may be benefits from the nipple shield delivery if breastmilk is proven to provide taste masking effects.”

**Response:** Thank you we have amended as suggested

**Minor point 4:** Please also add a statement to the above sentence that the bitter taste of some medicines might cause aversion of infants to breastfeeding.

**Response:** The following sentence has been added. ‘However, it would be important to determine the potential impact of taste, as the bitter taste of some medicines might cause aversion to breast feeding, and adjustment to formulations would be required to insure compliance.’

Sincerely,

Dr. Kathryn Beardsall
